# Pharmacological Activation of the EDA/EDAR Signaling Pathway Restores Salivary Gland Function following Radiation-Induced Damage

**DOI:** 10.1371/journal.pone.0112840

**Published:** 2014-11-19

**Authors:** Grace Hill, Denis Headon, Zoey I. Harris, Kenneth Huttner, Kirsten H. Limesand

**Affiliations:** 1 Department of Nutritional Sciences, University of Arizona, Tucson, Arizona, United States of America; 2 The Roslin Institute and Royal School of Veterinary Studies, University of Edinburgh, Edinburgh, United Kingdom; 3 Edimer Pharmaceuticals Inc., Cambridge, Massachusetts, United States of America; School of Medicine and Health Sciences, University of North Dakota, United States of America

## Abstract

Radiotherapy of head and neck cancers often results in collateral damage to adjacent salivary glands associated with clinically significant hyposalivation and xerostomia. Due to the reduced capacity of salivary glands to regenerate, hyposalivation is treated by substitution with artificial saliva, rather than through functional restoration of the glands. During embryogenesis, the ectodysplasin/ectodysplasin receptor (EDA/EDAR) signaling pathway is a critical element in the development and growth of salivary glands. We have assessed the effects of pharmacological activation of this pathway in a mouse model of radiation-induced salivary gland dysfunction. We report that post-irradiation administration of an EDAR-agonist monoclonal antibody (mAbEDAR1) normalizes function of radiation damaged adult salivary glands as determined by stimulated salivary flow rates. In addition, salivary gland structure and homeostasis is restored to pre-irradiation levels. These results suggest that transient activation of pathways involved in salivary gland development could facilitate regeneration and restoration of function following damage.

## Introduction

Approximately 40,000 new cases of head and neck cancer are diagnosed each year in the United States with five year survival rates around 64% [1]. Treatment for head and neck cancer typically involves surgical resection with subsequent radiation or chemoradiation therapy [2]. These necessary but tissue-nonspecific treatment modalities are associated with inadvertent and often clinically significant damage to surrounding normal tissues. Side effects include mucositis, xerostomia, dysphagia and malnutrition. Loss of saliva production in these patients predisposes them to periodontal disease, rampant caries, increased susceptibility to oropharyngeal candidiasis, changes in taste, and significant reductions in quality of life [3]–[5].

At a tissue level, salivary glands in close proximity to the tumor exhibit both acute and chronic responses to radiation damage. Depending upon the level of radiation exposure, chronic responses may last months, years and even become permanent after completion of radiation therapy [3]–[5]. A number of tissue pathologies have been reported in irradiated salivary glands including loss of acinar cells, focal inflammation, atrophy, vacuolization, and fibrosis [4]. As a result there are a significant number of patients who have completed their treatment regimen that continue to suffer from these side effects [6]. The currently available xerostomia treatment options are palliative at best and are not considered a long-term solution [3].

It has been postulated that tissue regeneration following injury may recapitulate normal development and utilize molecular signaling pathways active in organ morphogenesis [7]. The murine *Tabby* mutant is deficient in the key ectodermal signaling molecule ectodysplasin-A1 (EDA), which is required during prenatal development to trigger signal transduction from its receptor EDAR, thereby initiating development and morphogenesis of a range of structures, including hair follicles, teeth, and a number of glands, including salivary glands [8]. Absence of this signal results in generalized secretory gland hypoplasia including reduced salivary gland branching, weight, and secretory output [9]. This phenotype can be reversed in organ culture of embryonic *Tabby* salivary glands by administering exogenous EDA-A1 protein [10], [11]. *Tabby* is an animal model for the human ectodermal disorder XLHED (X-linked hypohidrotic ectodermal dysplasia), which is also caused by mutation of the *EDA* gene, and patients affected by XLHED have been demonstrated to have reduced salivary output.

A majority of salivary gland regeneration studies have focused on the ductal ligation protocol which involves surgical placement of a metal clip over the main excretory ducts of the submandibular salivary glands for 1–2 weeks followed by removal of the clip. Within 3–8 weeks of deligation, submandibular glands restore excretory function and glandular structure [12]. Embryonic-like branched structures can be detected during early time points following deligation (3–7 days) and these structures have been hypothesized to play a critical role in regeneration of acinar cells that were lost during the ligation phase [13], [14]. EDA/EDAR mediated signaling has been shown to promote branching morphogenesis during salivary and mammary gland development [9], [15]; however its role in regeneration is currently unknown.

Modulation of the EDAR pathway activity in adults has become possible with the development of pharmacological agents to stimulate signaling (recombinant EDA or monoclonal antibodies against EDAR) or to block endogenous EDA-A1 from binding to its receptor (monoclonal antibodies against EDA) [8]. mAbEDAR1 is an agonist monoclonal antibody that binds specifically to the extracellular domain of EDAR and activates the downstream EDA/EDAR signaling pathway [16]. In both mouse and dog models of XLHED, a single administration of mAbEDAR1 corrected the EDA-deficient hair and sweat gland phenotype demonstrating the functional equivalence of mAbEDAR1 and EDA ligand [16]. Based on the key role of EDA/EDAR signaling in embryonic salivary gland development, we hypothesized that mAbEDAR1 activation of this signaling pathway following head and neck irradiation could enhance salivary gland regeneration and reduce the clinically significant complications associated with xerostomia. We have used a mouse model to test this hypothesis, incorporating a 5 Gy single dose irradiation to the head and neck area, which includes major and minor salivary glands. We assessed the role of exogenously driven EDAR pathway activation in stimulating salivary gland regeneration and functional recovery from 3 days post-irradiation through day 90, similar to previous restoration models [17]. In this system we report that post-irradiation mAbEDAR1 therapy induces structural and functional restoration of salivary glands.

## Materials and Methods

### Mice Treatment

Experiments were conducted on 5 week old female FVB mice (Taconic Farms, Oxnard, CA). Mice were housed and treated in accordance with protocols approved by the University of Arizona Institutional Animal Care and Use committee (IACUC). On day zero, mice were anesthetized with an intramuscular injection of Ketamine/Xylazine (50 mg/kg/10 mg/kg respectively; Western Medical Supply, Arcadia, CA). Mice were then placed in a holding device, with the head and neck region exposed, while the remainder of the body was shielded with >0.6 mm of lead. Mice were irradiated with a single 5 Gy dose of radiation (day 0 of time course). On day four, a sub-group of untreated and irradiated mice received a single tail vein injection of mAbEDAR1 (5 mg/kg in PBS, Edimer Pharmaceuticals, Cambridge, MA) similar to a previous study using this compound [16]. The rationale for systemic (iv) administration of mAbEDAR included previous work on the functionality and half-life of mAbEDAR by this route, the feasibility of one injection reaching all major and minor salivary glands, and the greatest translatability to the clinical setting. The remaining untreated and irradiated mice received a vehicle (PBS) injection. All experiments were approved by IACUC.

### RNA isolation and RT/PCR Analysis

Total RNA was isolated from the parotid glands using the RNeasy isolation kit (Qiagen, Germantown, MD, USA) with DNase treatment and diluted to 200 ng/µL [18]. 5 µL of diluted RNA was added to 1 µL of Oligo(dT)_20_, 1 µL H_2_O and 1 µL of Annealing Buffer. This was heated to 65°C for 5 minutes, and cooled on ice for 1 minute. 10 µl of 2X First-Strand Reaction Mix and 2 µl of SuperScript III/RNAseOUT Enzyme Mix (Invitrogen, Carlsbad, CA, USA) were added and the reaction was incubated for 50 minutes at 50°C and 5 minutes at 85°C. cDNA was diluted with 80 µL of H_2_O for real-time PCR reaction. Each real-time PCR reaction contained 5 µl of diluted cDNA, 1 µl of each primer at 10 mM each, 12.5 µl SYBR Green (Qiagen), and nuclease-free water for a final volume of 25 µl. Forward and reverse primer (IDT, San Diego, CA) sequences are as follows: EDAR forward, TGTCCTCCATGCAGACCAG; EDAR reverse, GCATATCTGATAACCTCCTTTGG; S15 forward, ATCATTCTGCCCGAGATGGTG; S15 reverse, TGCTTTACGGGCTTGTAGGTG. Reactions were conducted in triplicate for each sample using the iQ5 Real-Time PCR Detection System (Bio-Rad, Hercules, CA). PCR conditions were, 95°C for 15 seconds, 54°C for 30 seconds, 72°C for 30 seconds for 40 cycles. Detection occurred during the 72°C step during each cycle. The data were analyzed using a 2^ΔΔCt^ method [19]. Thresholds cycle values were normalized to S15. Normalized values from 4 mice/group are represented as relative fold over untreated.

### Saliva Collections

Stimulated saliva collections were performed according to previously published reports [17], [20]–[25]. Mice received an intraperitoneal injection of carbachol 0.25 mg/kg (Sigma-Aldrich, St. Louis, MO) to stimulate salivary flow. Mice were then placed into a restraining device and saliva was collected for 5 minutes into weighed tubes on ice. Five minute collections on unanaesthetized mice were conducted according to a previously published study [26] and chosen to minimize the stress to the animals. Once completed, samples were then frozen on dry ice. Salivary flow rates (µg/min) were determined by calculating the change in collection tube weight (post-pre) divided by the collection time (5 min). The average µg/min value for the untreated animals was set to 100% (normalized value of 1) for each collection day. Each µg/min value for the experimental animals was divided by the average untreated µg/min value for each collection day to determine the percent change. Similar to our previous studies, no significant differences in body weight between the treatment groups were detected; therefore body weight is not used in the salivary flow rate calculation. Collections were performed on days 3, 14, 30, 60 and 90 post-irradiation with >16 mice per treatment.

### Tissue collection

Following anesthesia, salivary glands were removed, immediately fixed for 24 hours with 10% formalin (Fisher Diagnostics, Kalamazoo, MI) and then transferred to 70% ethanol. Tissue was embedded into paraffin, cut to 4 µm sections, and stained with hemotoxylin and eosin by the Histology Service Laboratory in the Department of Cellular and Molecular Medicine at the University of Arizona.

### Vacuole analysis

For each mouse, two images of hemotoxylin and eosin stained submandibular gland sections were collected at non-overlapping locations close to the center of the gland. Images were taken at 200X. Using ImagePro (Media Cybernetics, Silver Spring, MD) software, a subregion within each image was defined to avoid gland edges, large lumens, fissures, and large blood vessels. Within this area of interest each vacuole, defined as an unstained, acellular area greater than 7.35 µm in diameter, was outlined to determine its area. Unstained areas were not included for measurement if they were epithelially bounded (i.e. the unstained region was a lumen) or if they were long fissures related to the lobular structure of the gland. Clusters of vacuoles separated only by a thin strand of cytoplasm, and not separated by any cell nuclei, were measured as a single vacuole. Area data for vacuoles from a given image were summed using ImagePro software and used to determine the proportion of total area of interest that was represented by vacuoles. The values for the two images collected from each animal were averaged to yield a vacuole percentage for each animal and these results were then evaluated for each treatment group (N = 4/group). All vacuoles were in the acinar compartment and no vacuoles were found within the ductal compartment.

### Amylase staining

Serial sectioned unstained slides were incubated at 37°C for 30 minutes. Paraffin was removed though incubation in Histo-Clear (National Diagnostics, Atlanta, GA) followed by rehydration washes in 100%, 95%, 70%, 50% ethanol and deionized water. Peroxidases were neutralized with 0.3% H_2_O_2_ (Fisher Scientific, Fair Lawn, NJ). Slides in 10 mM citric acid (pH 6.0) were heated in a microwave for two 5 minute intervals then cooled for 20 minutes at room temperature. Slides were washed with PBS, blocked with 0.5% NEN (Boston, MA) at room temperature for 1 hour, and then incubated in anti-amylase primary antibody (1∶1000) overnight at 4°C (Sigma-Aldrich). Slides were washed and incubated in secondary antibody Anti-Rabbit AlexaFluor594 (1∶500) (Invitrogen) at room temperature for 1 hour, counterstained with DAPI, then mounted with 50% glycerol in 10 mM Tris-HCl (pH 8.0). Images were obtained using a Leica DM5500 Microscope System and captured with a Spot Pursuit 4 Megapixel CCD camera (Diagnostic Instruments, Inc., Sterling Heights, MI). Analysis was performed with ImagePro 6.3 software. Amylase-positive area was measured from twenty fields of view (FOV = 0.39 mm^2^) per mouse with 4 mice per treatment. Data are shown as the percent of amylase positive area to the total area of the parotid gland.

### PCNA staining

Serial sectioned unstained slides were incubated at 37°C for 30 minutes. Paraffin removal, rehydration, peroxidase neutralization and antigen retrieval were performed as described above for amylase staining. Slides were washed with PBS, blocked with goat serum from the ABC Rabbit Kit (Vector Laboratories, Burlingame, CA) and then incubated with anti-PCNA antibody (Santa Cruz Biotechnology) overnight at 4°C. Slides were washed and incubated in secondary antibody from the ABC Rabbit Kit. Color detection utilized DAB (Biogenex Laboratories, Fremont, CA) for 6 min per slide. Slides were counterstained using Harris hematoxylin (Sigma-Aldrich, St. Louis, MO), dehydrated, and mounted using Protocol Securemount (Fisher Scientific). Images were obtained using a Leica DM5500 and 4 megapixel Pursuit camera. PCNA-positive and total acinar cells in the parotid gland were counted manually from at least 3 fields per slide at 200X. Data are graphed as a percentage of PCNA positive acinar cells to total acinar cells from 4 mice per treatment.

### Statistical analysis

**Figure 1:**
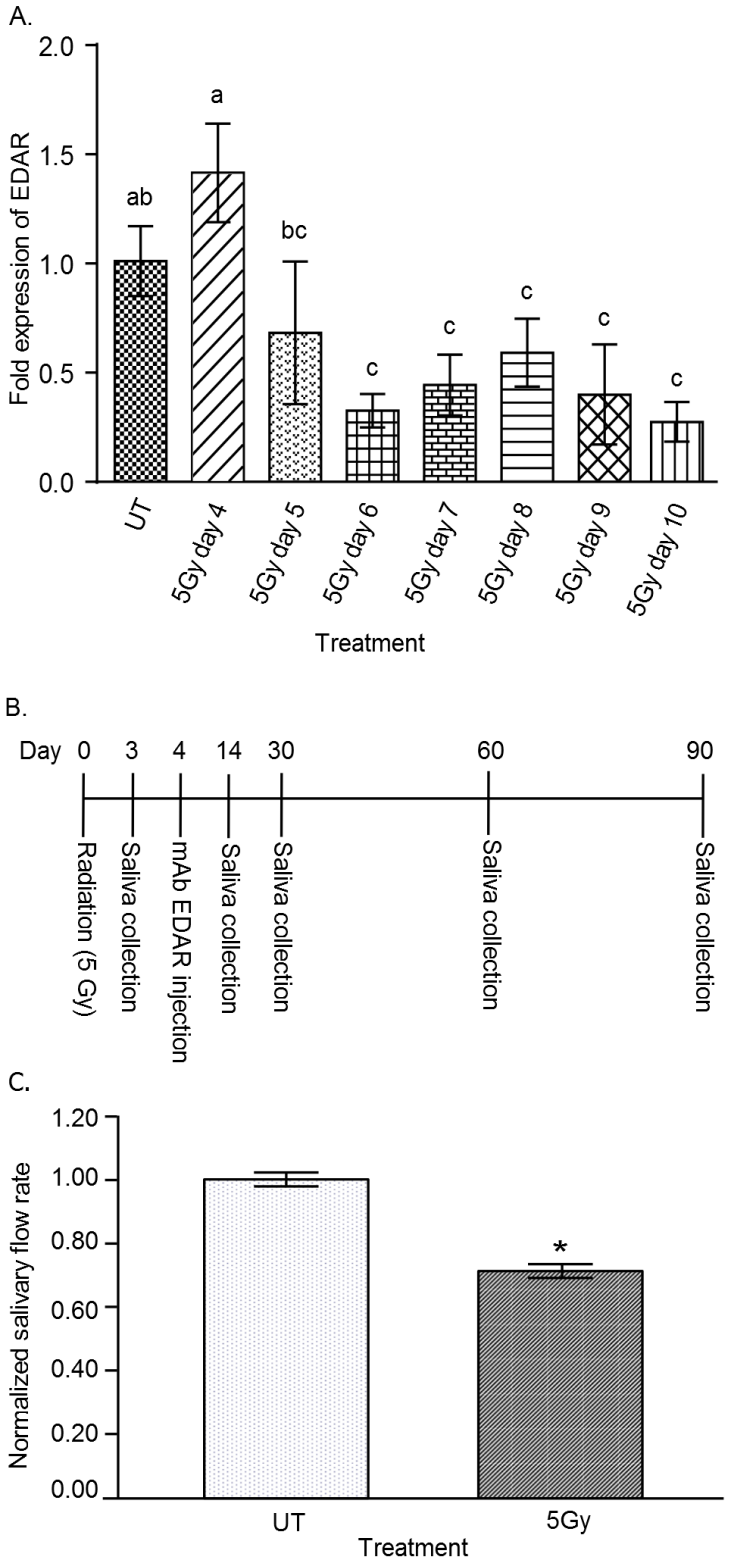
Analysis of *Edar* expression following radiation and experimental design of study. (A) On day 0 the head and neck region of mice was exposed to a single dose of 5 Gy radiation. *Edar* expression (mRNA) in parotid salivary glands was determined on days 4–10 post-radiation as described in the materials and methods and graphed as fold over unirradiated (UT). Significant differences (p<0.05) were determined using an ANOVA followed by a Bonferroni post-hoc test. Treatment groups with the same lower case letters are not significantly different from each other. N = 4 mice per group. (B) Experimental design timeline. (C) Three days post-radiation stimulated salivary flow rates were determined as described in the materials and methods. Irradiated flow rates were normalized to corresponding unirradiated (UT) controls. There were 60 animals in the control (UT) and 62 animals in the irradiated group (5Gy). Significant difference (p<0.05) was determined by t-test and designated by an asterisk (*). All error bars represent standard error of the mean (SEM).

Salivary flow rates were normalized by collection day to corresponding untreated group for each time point and analyzed by ANOVA with a Student-Newman-Keuls post-hoc test. For Figure 1C only, salivary flow rate comparison between unirradiated (UT) and irradiated (5Gy) was determined by t-test using Microsoft Excel. Real-time RT/PCR, vacuole area, amylase area and PCNA indices were analyzed by ANOVA with a Bonferroni post-hoc test. Statistical analysis of data and graph generation was completed using Graph-Pad software. All error bars represent standard error of the mean (SEM). Multiple comparison statistical differences are represented by lower case letters within individual graphs. Treatment groups with the same letters are not significantly different from each other. Therefore if a group is designated as “a” then it is statistically different from a group designated as “b” or “c”. If a group is designated as “ab” then it is not statistically different from a group designated as “a” or “b”.

## Results

### EDA receptor levels are maintained at day 4 and progressively decline at days 5–10 following radiation

Activation of the EDA/EDAR signaling pathway by mAbEDAR1 relies on endogenous expression of the EDA-A1 receptor (EDAR). We sought to determine the effect of radiation on *Edar* expression levels over a time period similar to previously published salivary restoration studies (days 4–10; Figure 1A). Multiple comparison statistics were completed to compare each time point and treatment groups with the same letters are not significantly different from each other. Four days after radiation treatment, *Edar* levels were not statistically different (both within statistical group “a”) from unirradiated controls (UT). At day 5, *Edar* levels began to decline (not statistically different from unirradiated controls or radiation day 4) and by radiation days 6–10 were significantly reduced at when compared to unirradiated controls. Therefore the experimental design (Figure 1B) utilized an injection of mAbEDAR1 on day 4, which is after the significant reductions in salivary flow rates (day 3, Figure 1C) but prior to reductions in *Edar* expression (Figure 1A).

### mAbEDAR1 administration following radiation restores stimulated salivary flow rates to pretreatment levels

**Figure 2:**
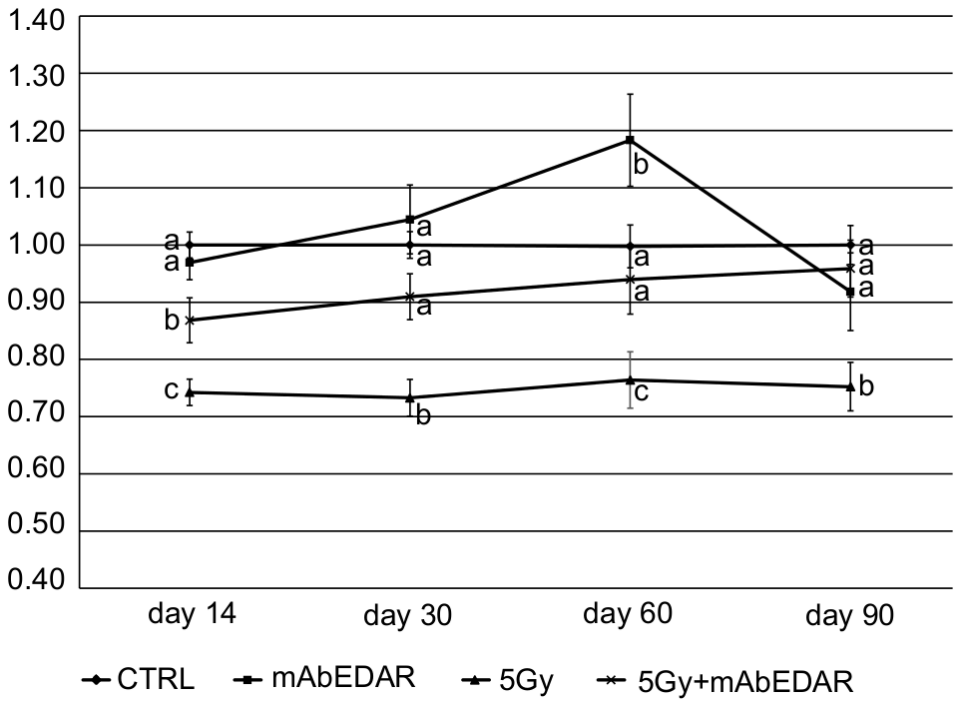
mAbEDAR1 administration following radiation restores stimulated salivary flow rates to pretreatment levels. On day 0, the head and neck region of mice was exposed to a single dose of 5 Gy radiation. On day 4, mice received a single dose injection of mAbEDAR or vehicle control. Stimulated salivary flow rates were determined on days 14, 30, 60, 90 post-irradiation. Flow rates (mean+/−SEM) were calculated by determining the average µg/min value for the unirradiated controls (CTRL) for each collection day followed by the percent change in individual experimental animals. Unirradiated controls averaged ∼27 µg/min across all time points and were set to 100% (normalized value of 1) for each time point. Significant differences (p<0.05) were determined using an ANOVA followed by a Student-Newman-Keuls post-hoc test. Treatment groups with the same lower case letters are not significantly different from each other. All error bars represent standard error of the mean (SEM). N>16 mice per group.

Once significant reductions in salivary flow rates were confirmed in irradiated animals (Figure 1C), mice were randomized into mAbEDAR1 or vehicle control treatment groups and respective injections were administered on day 4. Stimulated salivary flow rates were again determined at days 14, 30, 60 and 90 following radiation treatment (Figure 2) with multiple comparison statistics completed by time point (treatment groups with the same letters are not significantly different from each other). On average, untreated flow rates were ∼27 µg/min across all time points. At day 14, irradiated animals had significant reductions in stimulated salivary flow rates (statistical group “c”) when compared to unirradiated controls (CTRL, statistical group “a”). In contrast, animals receiving post-therapy mAbEDAR1 have improved stimulated salivary flow rates (statistical group “b”) that are significantly higher than irradiated animals, albeit the levels have not reached unirradiated controls. Following these animals from days 30–90 revealed that post-therapy mAbEDAR1 was able to restore stimulated salivary flow rates to untreated levels (both statistical group “a”) while irradiated animals continued to display reduced function. Treatment with mAbEDAR1 alone did not alter stimulated salivary flow rates at days 30 and 90; however salivary secretion was significantly higher in these animals at day 60. It is unclear why treatment with mAbEDAR alone resulted in elevated secretion at one intermediate time point (day 60), while it was similar to untreated controls at days 14, 30 and 90. Overall, these results suggest that post-therapy mAbEDAR1 is able to restore secretion in salivary glands that have been damaged by radiation.

### Increased vacuolization in irradiated salivary glands is reversed with post-therapy mAbEDAR1

**Figure 3:**
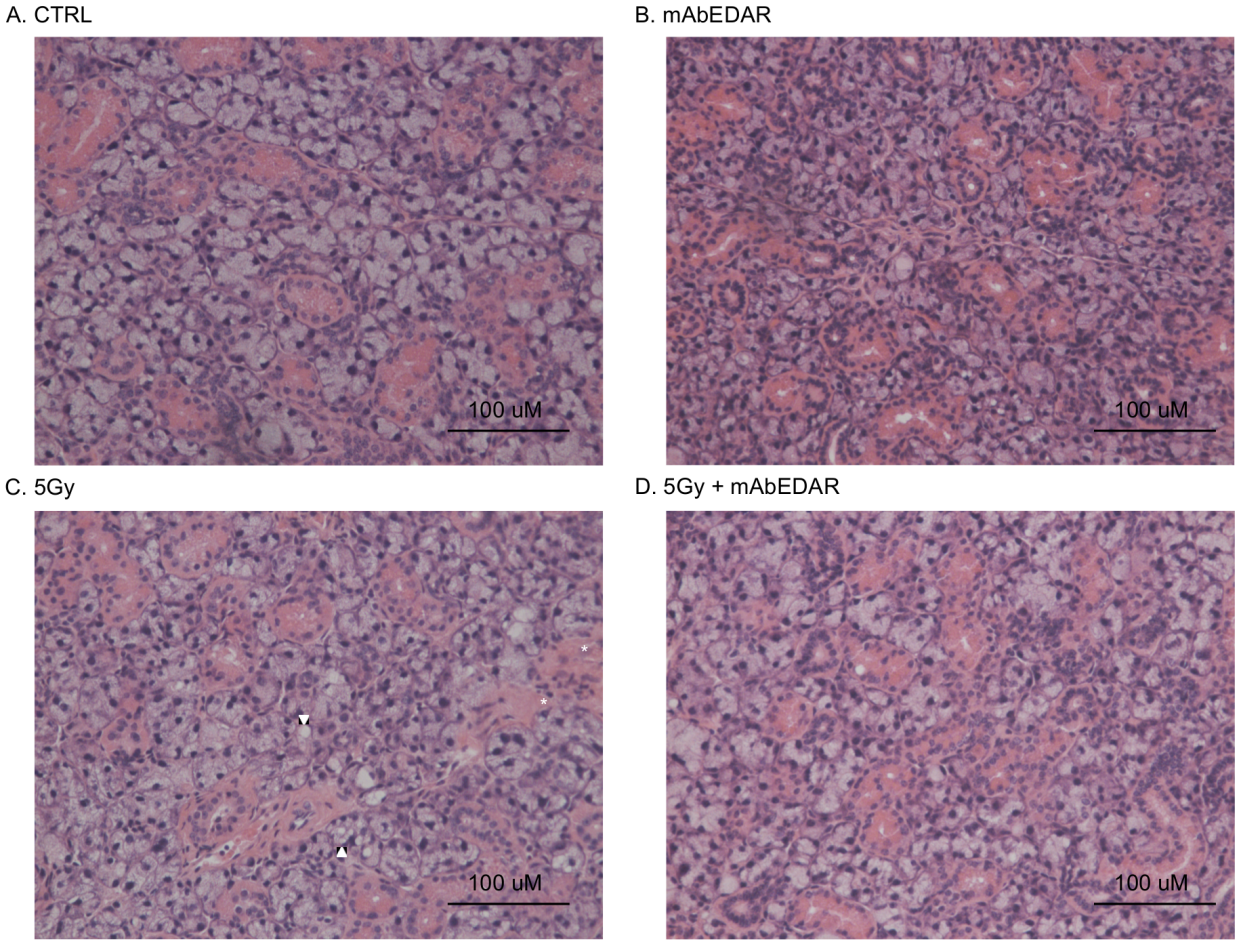
Histological analysis of salivary glands. Mice were treated as described in Figure 2 and submandibular salivary glands were collected at day 60 for histological analysis. Representative H&E images from unirradiated control (A), mAbEDAR1 alone (B), irradiated with 5Gy (C) and 5Gy+mAbEDAR are shown (D). Areas of focal inflammation denoted with asterisks and vacuoles are delineated by an arrowhead.

**Figure 4:**
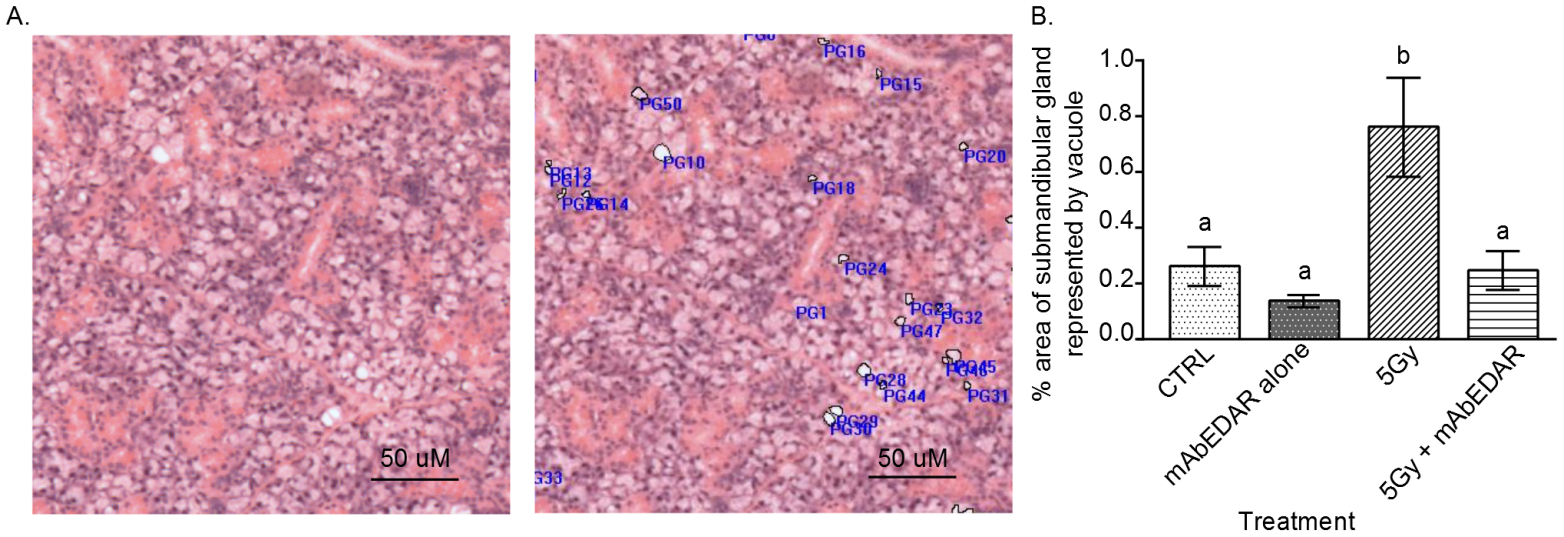
Increased vacuolization in irradiated salivary glands is reversed with post-therapy mAbEDAR1. H&E images from submandibular salivary glands were analyzed for cellular vacuoles as described in the materials and methods. Vacuoles were defined as an unstained, acellular area greater than 7.35 µm in diameter. (A) Representative annotated image of vacuole measurement compared to the unmarked image is shown. ImagePro software was utilized to calculate the area annotated as a vacuole compared to the total area in the image. (B) Quantification of the percent area occupied by cellular vacuoles (mean+/−SEM) is graphed. Significant differences (p<0.05) were determined using an ANOVA followed by a Bonferroni post-hoc test. Treatment groups with the same lower case letters are not significantly different from each other. All error bars represent standard error of the mean (SEM). N = 4 mice per group.

Previously described histological changes in salivary glands following radiation consist of focal inflammation, atrophy and fibrosis [4]. Analysis of H&E sections from the current study revealed similar histological alterations as previous reports (pathologic features noted with arrowheads and asterisks in Figure 3 and data not shown) as well as the presence of cellular vacuoles in irradiated sections of the submandibular salivary gland. Vacuoles were defined as an unstained, acellular area greater than 7.35 µm in diameter that did not denote a lumen and were outlined to determine their aggregate area (representative annotated image in Figure 4A). The most prominent differences were detected at day 60 post-irradiation; therefore this time point was selected for further analysis. The percent area occupied by vacuoles (combined annotated area/total area) was significantly higher in irradiated submandibular glands when compared to unirradiated controls (Figure 4B). Importantly, the percent area with vacuoles in animals receiving mAbEDAR1 following radiation was not different from untreated controls. These data suggest that radiation induces persistent histological changes in salivary gland structure that is repaired in animals treated with post-irradiation mAbEDAR1.

### Restoration of amylase positive area in parotid glands of mice treated with post-therapy mAbEDAR1

**Figure 5:**
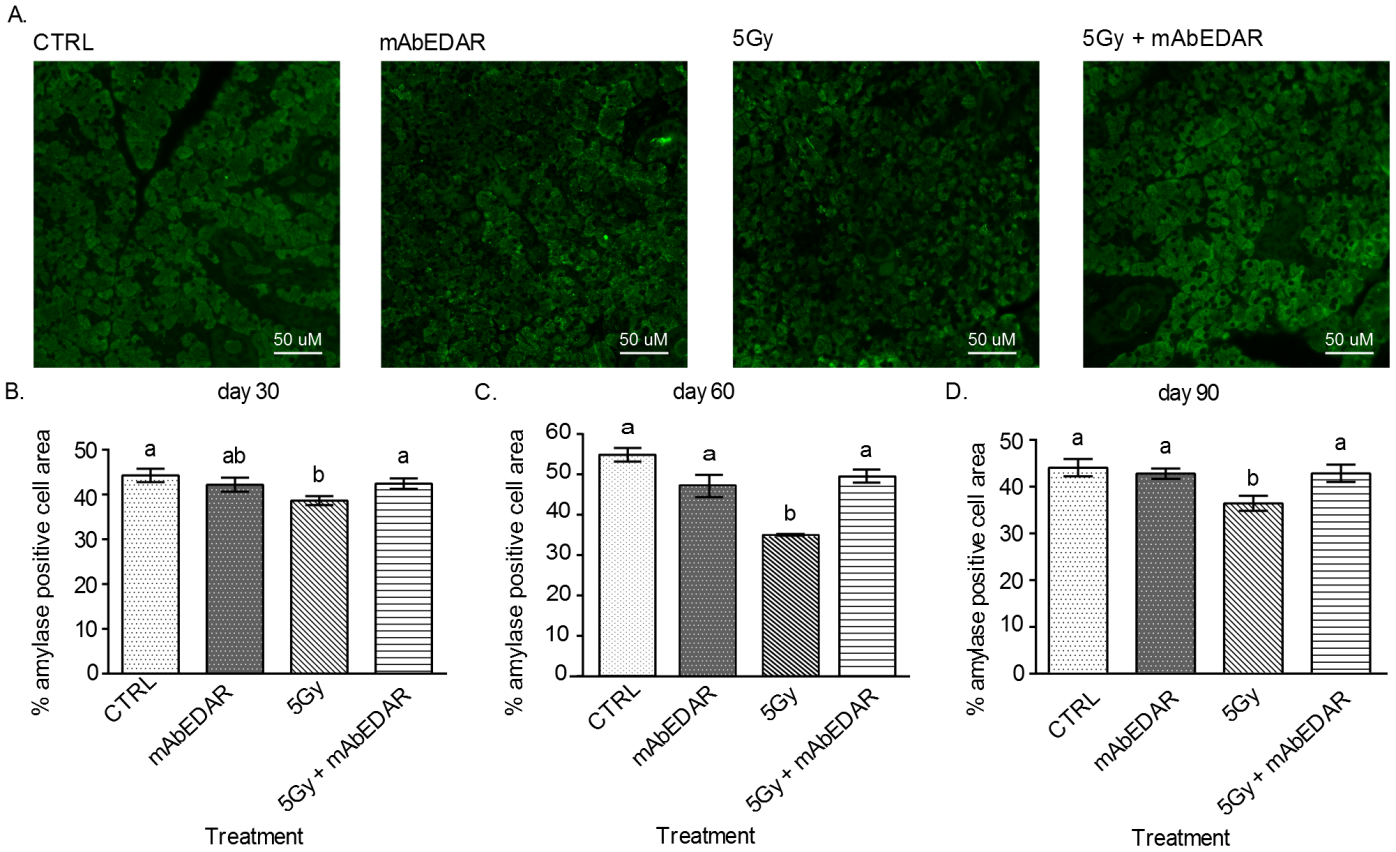
Restoration of amylase positive area in parotid glands of mice treated with post-therapy mAbEDAR1. Mice were treated as described in Figure 2 and parotid salivary glands were collected at days 30, 60, and 90 for analysis of amylase area. (A) Representative images from each treatment group are shown. Quantification of the mean area of parotid tissue staining positive for amylase was performed as described in the materials and methods at days 30 (B), 60 (C) and 90 (D). Significant differences (p<0.05) were determined using an ANOVA followed by a Bonferroni post-hoc test. Treatment groups with the same lower case letters are not significantly different from each other. All error bars represent standard error of the mean (SEM). N = 4 mice per group.

Saliva production requires the movement of water as well as the production of numerous salivary proteins [27]. Amylase is a salivary protein made by parotid salivary glands that is frequently reduced following radiation treatment [17], [25], [28]. Therefore we determined the area of the parotid gland that had restored amylase protein production through morphometric analysis at days 30, 60 and 90 (Figure 5). Irradiated parotid glands had significant reductions in the area expressing amylase when compared to untreated controls at each time point evaluated. In contrast, animals receiving mAbEDAR1 following radiation had restored amylase expression in the parotid gland and there was no statistical difference from untreated controls at each time point evaluated. Similarly, animals treated with mAbEDAR1 alone did not display significant changes in amylase area in the parotid gland at each time point evaluated. These data suggest that post-therapy mAbEDAR1 restores amylase protein production in parotid glands damaged by radiation.

### Reduced compensatory proliferation in mice treated with post-therapy mAbEDAR1

**Figure 6:**
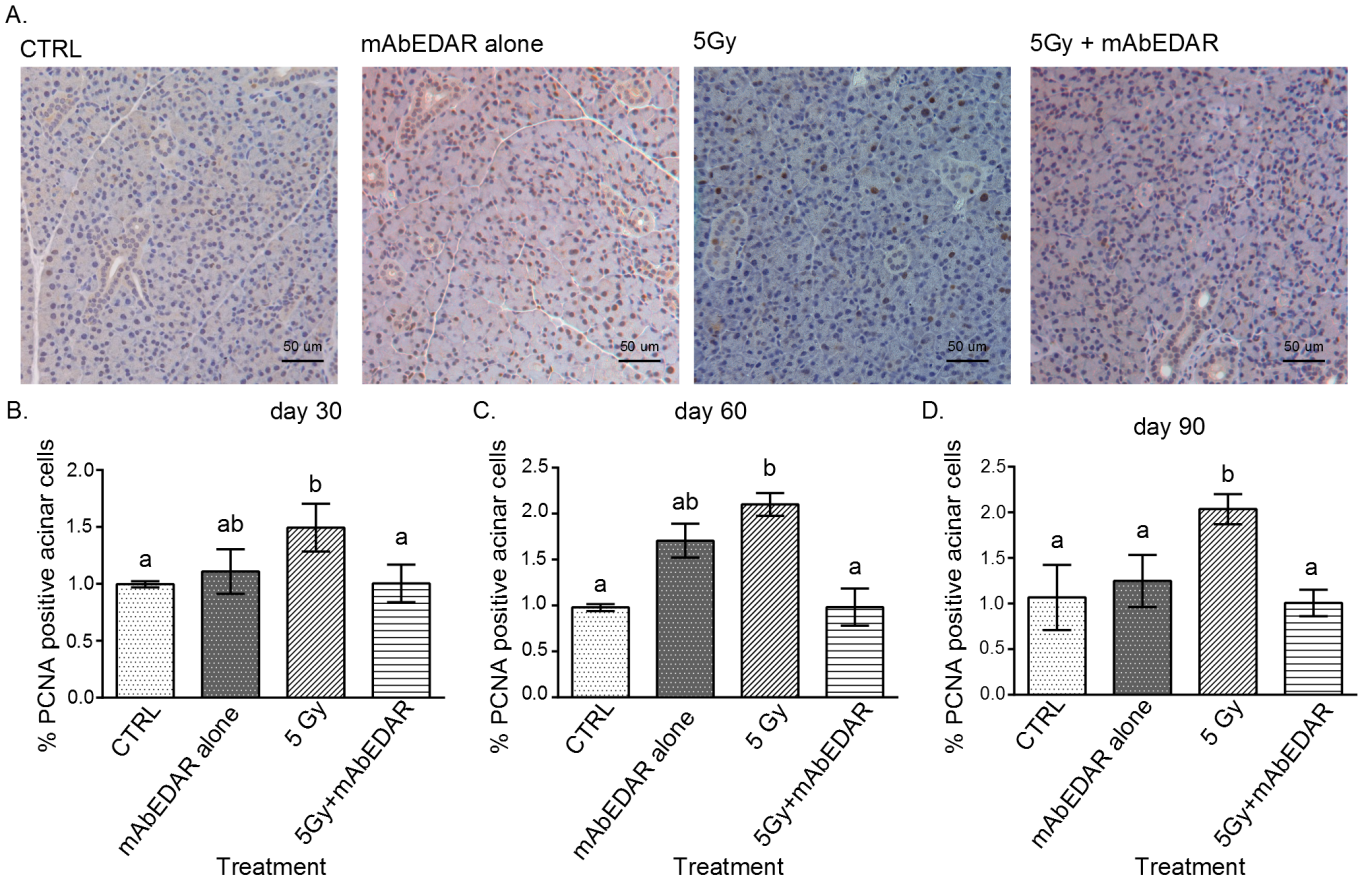
Reduced compensatory proliferation in mice treated with post-therapy mAbEDAR1. Mice were treated as described in Figure 2 and parotid salivary glands were collected at days 30, 60, and 90 for analysis of PCNA levels. (A) Representative images from each treatment group are shown. The total number of PCNA positive cells in the acinar compartment was graphed as a percentage of total number of cells in the acinar compartment at days 30 (B), 60 (C) and 90 (D). Significant differences (p<0.05) were determined using an ANOVA followed by a Bonferroni post-hoc test. Treatment groups with the same lower case letters are not significantly different from each other. All error bars represent standard error of the mean (SEM). N = 4 mice per group.

Glandular homeostasis requires a balance between proliferation and differentiation. Radiation damage causes apoptosis of salivary acinar cells [20], [29] and loss of these cells induces a proliferation response in the surrounding cells [17]. This compensatory proliferation response is intended to replace the cells lost to apoptosis [30]; however prolonged increases in proliferation are correlated with reductions in salivary flow rates [17]. Therefore we determined the level of proliferation through PCNA staining and quantification of the number of positive cells in the acinar compartment at days 30, 60 and 90 (Figure 6). At each time point evaluated, irradiated animals have significant increases in the number of PCNA positive cells when compared to untreated controls. In contrast, animals receiving mAbEDAR1 following radiation have reduced numbers of PCNA positive cells which does not differ from untreated controls each time point evaluated. Animals treated with mAbEDAR1 alone exhibited more variation in proliferation levels; however this group was not statistically different from untreated controls at each time point evaluated (mAbEDAR statistical group “ab” is not different from statistical group “a”). These results suggest that salivary gland homeostasis, as well as histological structure and glandular function, are restored in animals treated post-therapy with mAbEDAR1.

## Discussion

Due to the chronic effects of radiation on salivary gland function, there are numerous patients who have completed their anti-cancer therapy yet still suffer the side effects of that therapy. Currently there are few approved therapies that can substantially improve the quality of life for these individuals [3]. In this preclinical study we have investigated a new therapeutic for its potential use in restoring salivary gland function following radiotherapy. Utilization of mAbEDAR1 in animals with radiation-induced loss of function led to an improvement in physiological function of the salivary glands at day 14 and a complete restoration of function by day 30 (Figure 2). This improvement in function was maintained to 90 days post radiation. Restoration of salivary gland function has been previously described utilizing gene therapy with Adenoviral vectors or growth factors (IGF1 or KGF) [6], [17], [31], [32]. Adenoviral restoration of salivary gland function has primarily focused on increasing expression of a water channel (aquaporin 1) via local injection into salivary glands [32]. This results in improved salivary output within three days and subsequent studies have extended this effect in a phase I clinical trial [6]. The results in the current study are similar to a previous study with post-radiation injections of Insulin-like Growth Factor 1 (IGF1). Post therapy injections of IGF1 resulted in improved salivary output at day 30 and complete restoration by day 60 [17]. Post therapy injection of mAbEDAR resulted in an expedited response with improved salivary output at day 14 and complete restoration by day 30 (Figure 2). In contrast, post-radiation injections of Keratinocyte Growth Factor (KGF) did not show improvement in salivary output at day 30 [31]. Importantly, mAbEDAR1 was administered through a single systemic injection while IGF1 was given over multiple injections. The effectiveness of a single administration of mAbEDAR1 may be due to the long half-life of this antibody, estimated at approximately 11 days *in vivo* [16]. Thus it is likely that sustained stimulation of the EDAR pathway was achieved in these experiments and the beneficial effect of treatment on salivary function persists beyond the time of plasma antibody clearance, as indicated by the sustained restoration measured at 90 days post-irradiation. While the objective measurement of salivary output does not always correlate with the subjective symptom of xerostomia [3], a recent clinical trial examining the re-expression of aquaporin channels following radiation suggests that increasing fluid movement has a positive effect on xerostomia [6].

The presence of cytoplasmic vacuoles has been previously reported in both parotid and submandibular salivary glands [33]–[36]. Early ultrastructural analysis using electron microscopy of irradiated parotid glands detected small vacuoles four days after treatment [33]. Most studies have reported the histological presence of vacuoles at acute time points (up to 10 days); however a few studies looked at more chronic time points (60–90 days) [34]–[36]. We have extended this analysis by quantifying the percentage of salivary gland area occupied by vacuoles following radiation (Figure 4). Importantly, injection of mAbEDAR1 post-radiation was able to reduce the aggregate area with vacuoles in the submandibular glands and restore the overall histological structure to that of an unirradiated state (Figure 4). The presence of vacuoles has also been reported upon acute starvation of rats with small vacuoles appearing in salivary glands at 24 hours that increase in size at 72 hours [37]. However in the current study, irradiated animals continue to eat and do not lose more than 10% body weight so reductions in food intake do not appear to explain the chronic presence of these vacuoles.

Saliva is a complex fluid and requires the movement of water through aquaporin channels and the production of saliva proteins by the acinar cells [27]. Therefore, alterations to the differentiation state of the gland can adversely affect these crucial functions. Clinically, radiation exposure to salivary glands leads to a loss of amylase production and secretion, which has been recapitulated in animal models [17], [25], [28]. In addition, the loss of salivary acinar cells following radiation treatment induces a compensatory proliferation response to replace these cells [38]–[40]. However if proliferation remains elevated, it correlates with chronic loss of salivary gland function [17]. Since mAbEDAR1 was able to restore salivary secretion, it was important to validate that glandular homeostasis was also restored through amylase area analysis and proliferation indices. Similar to previously published models, radiation treatment led to reductions in amylase area (Figure 5) and increases in proliferation indices (Figure 6), which are indicative of an overall decrease in differentiation. In contrast, treatment with mAbEDAR1 following radiation led to improved amylase area and decreased compensatory proliferation which is indicative of restored homeostasis. Relatively few studies have confirmed improvements in secretion with salivary protein production. The effects of mAbEDAR1 on glandular homeostasis are similar to the model of IGF1 restoration [17]. This suggests that therapies which target improving the balance between differentiation and proliferation could restore function in this population.

Mouse models with defects in EDA or EDAR have both acinar and ductal phenotypes suggesting this pathway is important to a majority of salivary cells. In situ hybridization or immunohistochemistry detection of EDA/EDAR has been conducted on developmental time points in the submandibular gland and have shown the presence of EDA/EDAR in ducts and terminal end buds [9], [10]. In addition, it has been previously demonstrated that EDA is required for epithelial differentiation of the salivary gland [9]. Since acinar cells undergo apoptosis at acute time points (<48 hr) following radiation, it is possible that the EDA pathway facilitates the differentiation step of new acinar cells during regeneration.

This is the first study to our knowledge that utilizes pharmacological activation of a developmentally relevant pathway in reversing radiation damage to salivary glands. EDAR signaling has been shown to promote epithelial expansion and branching in cultured embryonic salivary glands [9], [10], and, *in vivo*, high level EDAR function in transgenic mice results in more highly branched adult salivary glands [41]. Embryonic-like branched structures also appear to be important in regeneration of adult mouse salivary glands following ductal ligation/deligation [13], [14] and these structures could be responsive to EDAR stimulation. However, significant reductions in *Edar* expression several days after irradiation (Figure 1A) could confound the ability of salivary glands to regenerate by limiting the presence of these regenerative branching structures. In addition to its effects on branching morphology, loss of EDAR function also leads to impaired cellular differentiation and duct formation [9]. Administration of mAbEDAR following radiation resulted in improved histological structure and amylase area (differentiation marker) suggesting a role in tissue pathology recovery and facilitation of differentiation during regeneration. (Figures 3 and 5). It would be of interest to determine whether there are commonalities in the cellular and molecular mechanisms of action of EDAR stimulation in the context of regenerating, as opposed to developing, salivary glands. It has been previously shown that EDAR signaling is not necessary for initiation of salivary glands which suggests this pathway may not affect stem cells during development [9]; however future studies could address the involvement of EDAR signaling in repair and regeneration. While exploratory in nature, these results highlight the potential for novel therapeutic paradigms to address a significant unmet medical need in the field of head and neck oncology.
